# The short mRNA isoform of the immunoglobulin superfamily, member 1 gene encodes an intracellular glycoprotein

**DOI:** 10.1371/journal.pone.0180731

**Published:** 2017-07-07

**Authors:** Ying Wang, Emilie Brûlé, Tanya Silander, Beata Bak, Sjoerd D. Joustra, Daniel J. Bernard

**Affiliations:** 1 Centre for Research in Reproduction and Development, Department of Pharmacology and Therapeutics, McGill University, Montreal, Quebec, Canada; 2 Department of Pediatrics, Leiden University Medical Center, Leiden, The Netherlands; Wayne State University, UNITED STATES

## Abstract

Mutations in the immunoglobulin superfamily, member 1 gene (*IGSF1/Igsf1*) cause an X-linked form of central hypothyroidism. The canonical form of IGSF1 is a transmembrane glycoprotein with 12 immunoglobulin (Ig) loops. The protein is co-translationally cleaved into two sub-domains. The carboxyl-terminal domain (CTD), which contains the last 7 Ig loops, is trafficked to the plasma membrane. Most pathogenic mutations in *IGSF1* map to the portion of the gene encoding the CTD. *IGSF1/Igsf1* encodes a variety of transcripts. A little studied, but abundant splice variant encodes a truncated form of the protein, predicted to contain the first 2 Ig loops of the full-length IGSF1. The protein (hereafter referred to as IGSF1 isoform 2 or IGSF1-2) is likely retained in most individuals with *IGSF1* mutations. Here, we characterized basic biochemical properties of the protein as a foray into understanding its potential function. IGSF1-2, like the IGSF1-CTD, is a glycoprotein. In both mouse and rat, the protein is N-glycosylated at a single asparagine residue in the first Ig loop. Contrary to earlier predictions, neither the murine nor rat IGSF1-2 is secreted from heterologous or homologous cells. In addition, neither protein associates with the plasma membrane. Rather, IGSF1-2 appears to be retained in the endoplasmic reticulum. Whether the protein plays intracellular functions or is trafficked through the secretory pathway under certain physiologic or pathophysiologic conditions has yet to be determined.

## Introduction

Loss-of-function mutations in the immunoglobulin superfamily, member 1 gene (*IGSF1/Igsf1*) cause central hypothyroidism in humans (OMIM #300888) and mice [1–3]. IGSF1 is abundantly expressed in the developing and adult pituitary gland [3–5]. According to observations in both humans and mice, IGSF1-deficiency is associated with impaired hypothalamic thyrotropin-releasing hormone (TRH) stimulation of thyroid-stimulating hormone (TSH) synthesis and/or secretion by thyrotrope cells of the anterior pituitary [2, 3, 6, 7]. IGSF1’s normal function in cells and how its absence leads to impaired TRH action are presently unknown.

The *IGSF1/Igsf1* gene encodes several mRNA transcripts in a tissue-specific manner [4, 8–10]. The most thoroughly characterized transcript derives from 20 exons and encodes a large transmembrane glycoprotein of 12 C2-type immunoglobulin (Ig) loops. This protein is co-translationally cleaved into N- and C-terminal domains (NTD and CTD) [11]. According to *in vitro* analyses, the CTD traffics to the plasma membrane, whereas the NTD is retained in the endoplasmic reticulum (ER). The CTD contains the last 7 of 12 Ig loops (from the full-length IGSF1), a transmembrane domain, and a short cytoplasmic tail, and is generally regarded as the functional part of the protein. This concept derives from at least two observations. First, most intragenic *IGSF1* mutations map to the part of the gene encoding the CTD [1–3, 12–18]. Second, the CTD can be produced from a mRNA isoform derived from an intronic promoter, at least in mouse [4, 7, 11]. Therefore, expression of the NTD is not required for expression or proper membrane trafficking of the CTD. Nonetheless, the 5 Ig loop-containing NTD is conserved across mammalian species, suggesting that it may have currently unappreciated functions.

During the initial characterization of *Igsf1* mRNAs in rats, a truncated but abundant transcript was identified in both pituitary and testis [10]. It was cloned from human and murine pituitaries shortly thereafter [4, 19]. This mRNA variant shares 5’ sequence through the first 5 exons with the full-length *IGSF1/Igsf1*. However, it retains part of the 5^th^ intron and protein translation terminates within this sequence. The open-reading frame of this variant (hereafter IGSF1 isoform 2 or IGSF1-2) is predicted to encode the first 2 of the 12 Ig loops in the full-length IGSF1. As the protein contains an N-terminal signal peptide but lacks a transmembrane domain, it was predicted to be secreted [10], but this has not been demonstrated experimentally. Indeed, to date, there are no published reports on characterization of IGSF1-2. Here, we investigated whether IGSF1-2 is a secreted protein.

## Materials and methods

### Cell culture and transfections

CHO cells (ATCC CCL-61; provided by P. Morris, Population Council, New York, NY) were cultured in DMEM/Ham’s F-12 (319-075-CL, Wisent Inc., Saint-Jean-Baptiste, Québec) supplemented with 10% fetal bovine serum (FBS; 12483–020, Gibco, Burlington, Ontario). Cells were seeded at a density of 4x10^5^ cells/well in 6-well plates (3516, Costar, Corning, NY) and transfected with 16 μl Lipofectamine 2000 (11668019, ThermoFisher, Burlington, Ontario) and 4 μg plasmid DNA. HEK293 cells (ATCC CRL-1573; provided by T. Hébert, McGill University, Montréal) were maintained in DMEM (319-005-CL, Wisent Inc.) supplemented with 7% (v/v) FBS. Cells were seeded at a density of 4x10^6^ per 10-cm dish (08-772-23, ThermoFisher) and transfected the following day with 21 μg polyethylenimine (PEI; 23966, Polysciences Inc., Warrington, PA) and 7 μg plasmid DNA. HepG2 cells (ATCC HB-8065) were cultured in EMEM (320-026-CL, Wisent Inc.) supplemented with MEM nonessential amino acid solution (321-011-CL, Wisent Inc.) and 10% (v/v) FBS. Cells were seeded at a density of 8 x10^6^ per 10-cm dish and transfected the following day with 42 μl Lipofectamine 2000 and 7 μg plasmid DNA. All cells were grown in a humidified environment at 37°C with 5% CO_2_.

### Constructs

The rat IGSF1-2 expression vector was generated by PCR amplifying the coding sequence of *Igsf1-2* from a rat testis cDNA library clone and ligating it into the *Kpn*I and *Apa*I sites of pcDNA4/Myc-HIS-A (Invitrogen, Carlsbad, CA). All PCR primer sequences are provided in Table 1. The murine IGSF1-2 expression vector was generated through a three-step process. First, the coding sequence was PCR amplified from adult mouse pituitary cDNA and ligated into the *Hind*III and *Xho*I sites of pcDNA3.0. Two additional amplification reactions were performed starting with this plasmid to enable in-frame ligation into pcDNA4/Myc-HIS-A. Asn43Gln (N43Q) and N44Q mutations were introduced into the murine and rat IGSF1-2 expression vectors, respectively, using the QuikChange protocol (Agilent Genomics, Santa Clara, CA). The transthyretin (*Ttr*) coding sequence was PCR amplified from murine liver cDNA and ligated into the *Hind*III and *Xba*I sites of pcDNA4/Myc-HIS-A. The BMPR1A-Myc expression construct was previously described [20]. All constructs were confirmed by Sanger sequencing (Genome Québec, Montréal, Québec).

**Table 1:** Primers used for cloning and mutagenesis.

Plasmid	Sense	Antisense
r*Igsf1-2*	AGGCCGGTACCGGCTAGAGTGA	TTCGAAGGGCCCACCAGCCATGATCCCTGGGAT
m*Igsf1-2*	ACGGAAGCTTCTAAGCCATGATGCTTCGGACC	ACGGCTCGAGGTTTTGCTCAACCAGCCATGA
*mIgsf1*-2 GC insert	TCCCAGGGATCATGGCTGCGGTTGA	GGTTTTGCTCAACCGCAGCCATGATC
*mIgsf1*-2 *Xho*I site	GGGATCATGGCTGCGCTCGAGCAAA	CTCGAGGTTTTGCTCGAGCGCAGCCA
m*Igsf1-2* N43Q	CAGGCCCCTTGGGAGCAGATCACACTCTGGTGC	GCACCAGAGTGTGATCTGCTCCCAAGGGGCCTG
*rIgsf1-2* N44Q	GCACCAGAGTGTGATTTGCTCCCAAGGGGCCTG	CAGGCCCCTTGGGAGCAAATCACACTCTGGTGC
m*Ttr*-Myc/His	CCCAAGCTTGACAGGATGGCTTCCCTTCGA	GCTCTAGAATTCTGGGGGTTGCTGACGAC

### Deglycosylation and immunoblotting

Deglycosylation and immunoblotting were performed as previously described [3]. Briefly, protein lysates were prepared from cultured cells 24 h post-transfection using RIPA buffer (150 mmol/L NaCl, 50 mmol/L NaF, 10 mmol/L NaPO_4_, 2 mmol/L EDTA, 1% (v/v) NP-40, 1% (w/v) sodium deoxycholate, 0.1% (v/v) SDS) containing protease inhibitors (2 mg/ml aprotinin, 2 mg/ml leupeptin, 1 mg/ml pepstatin A, and 0.2 mol/L PMSF). For deglycosylation assays, protein lysates were denatured at 100°C for 10 min and deglycosylated by incubating with 50U EndoH (P0702S) or PNGaseF (P0704S, New England Biolabs, Whitby, Ontario) for 2 h at 37°C. For immunoblotting, 25 μg protein samples were denatured with Laemmli Buffer containing 2% (v/v) β-mercaptoethanol for 10 min at 70°C, resolved by SDS-PAGE on 14% (v/v) Tris-glycine polyacrylamide gels, and transferred to Protran nitrocellulose membranes (NBA083C001EA, Perkin Elmer, Waltham, MA). Membranes were blocked in Tris-buffered saline Tween [TBST; 150 mmol/L NaCl, 10 mmol/L Tris (pH 8.0), 0.05% (v/v) Tween 20] containing 5% (w/v) non-fat milk, incubated with primary antibody overnight at 4°C, washed with TBST, incubated for 2 h with secondary antibody, and washed again with TBST. The following antibodies were used at the indicated concentrations: monoclonal anti-mouse c-Myc (1:500, M5546, Sigma-Aldrich, St. Louis, MO), polyclonal rabbit anti-mouse IGSF1 (1:1000; previously described [11]), and HRP-conjugated goat anti-mouse (1:5000, BioRad, Mississauga, Ontario). Bands were visualized using ECL Western Lightning Plus (NEL105001EA, Perkin-Elmer, Boston, MA) and exposed to HyBlot CL autoradiography film (E3018, Denville Scientific Inc., Metuchen, NJ).

### Protein purification

Twenty-four h post transfection, culture medium was collected and cell debris removed by centrifugation (10 min at 4000 rpm). HisPur Cobalt Resin (89964, ThermoFisher) was washed with PBS and then added to the medium. The resin-medium mixture was incubated overnight at 4°C with rotation. Resin was allowed to settle by gravity, supernatant was removed, and the resin was washed with 10 mmol/L imidazole (I5513, Sigma Aldrich) in PBS. Bound proteins were eluted with 250 mmol/L imidazole in PBS for 5 min at 95°C. Four μg protein samples were resolved on 14% (v/v) Tris-glycine polyacrylamide gels and immunoblotted as described above.

### Cell surface biotinylation

Transfected HEK293 cells were washed with PBS and incubated with EZ-link Sulfo-NHS-LC-Biotin (21335, Pierce, Nepean, Ontario) diluted to 0.5 mg/mL in PBS for 30 min at 4°C. Cells were washed with PBS containing 100 mmol/L glycine and lysed in RIPA buffer as described above. Supernatant was incubated overnight at 4°C with EZview Red Anti-c-Myc Affinity gel (E6654, Sigma Aldrich) and eluted with Laemmli buffer containing 2% (v/v) β-mercaptoethanol. Blotting proceeded as described above; however, the membrane was blocked with TBS-T containing 5% (w/v) bovine serum albumin (BSA) and incubated with Vectastain ABC Elite as per manufacturer’s instructions (PK-6100, Vector Laboratories, Burlingame, CA) before being exposed to film.

### Immunofluoresence

Circular coverslips (12-545-80, Fisher) were placed in 24-well plates (CLS3527, Costar) and then coated with diluted 2.6X Matri-gel (356234, Corning). Next, 3x10^4^ HEK293 cells were seeded per well in DMEM supplemented with 7% (v/v) FBS and transfected the following day in DMEM with 1 μg of DNA and 3 μg of PEI for 5 h. Cells were then refed with complete medium. Approximately, 24 h post-transfection, cells were fixed with 4% (w/v) paraformaldehyde (PFA; P6148, Sigma-Aldrich) for 2 min and washed with PBS three times. Some cells were treated with PBS containing 0.5% (v/v) Triton X-100 to permeabilize the membrane; other cells were left intact. Cells were blocked for 1 h with PBS containing 15% (v/v) goat serum (053–110, Wisent Inc.). For permeabilized cells, the blocking solution also contained 0.2% (v/v) Triton X-100. Cells were incubated with monoclonal mouse anti-c-Myc Ab (Sigma M5546; 1:500) overnight. The following day, cells were washed with PBS, incubated with Alexa Fluor 488 rabbit anti-mouse (A-21121, ThermoFisher Scientific; 1:500) for 1 h and washed again with PBS. Coverslips were mounted on slides using Prolong Gold Antifade reagent with DAPI (1266174, ThermoFisher Scientific). Confocal images were captured as Z-stacks with a Leica SP8 multiphoton microscope with a 63X objective (N.A. 1.4). Double-labeling for Myc and GRP-78/BiP (Abcam, ab21685; 1:500) was performed similarly with a few exceptions: 1) cells were seeded at a density of 5x10^4^, 2) fixation in 4% paraformaldehyde was reduced to 2 min, 3) incubation in primary antibodies was for 1 h at room temperature rather than overnight, and 4) the secondary antibodies were Alexa Fluor 488 donkey anti-mouse (A-21202, ThermoFisher Scientific; 1:500) and Alexa Fluor 594 donkey anti-rabbit (A-21207, ThermoFisher Scientific; 1:500).

## Results and discussion

**Fig 1:**
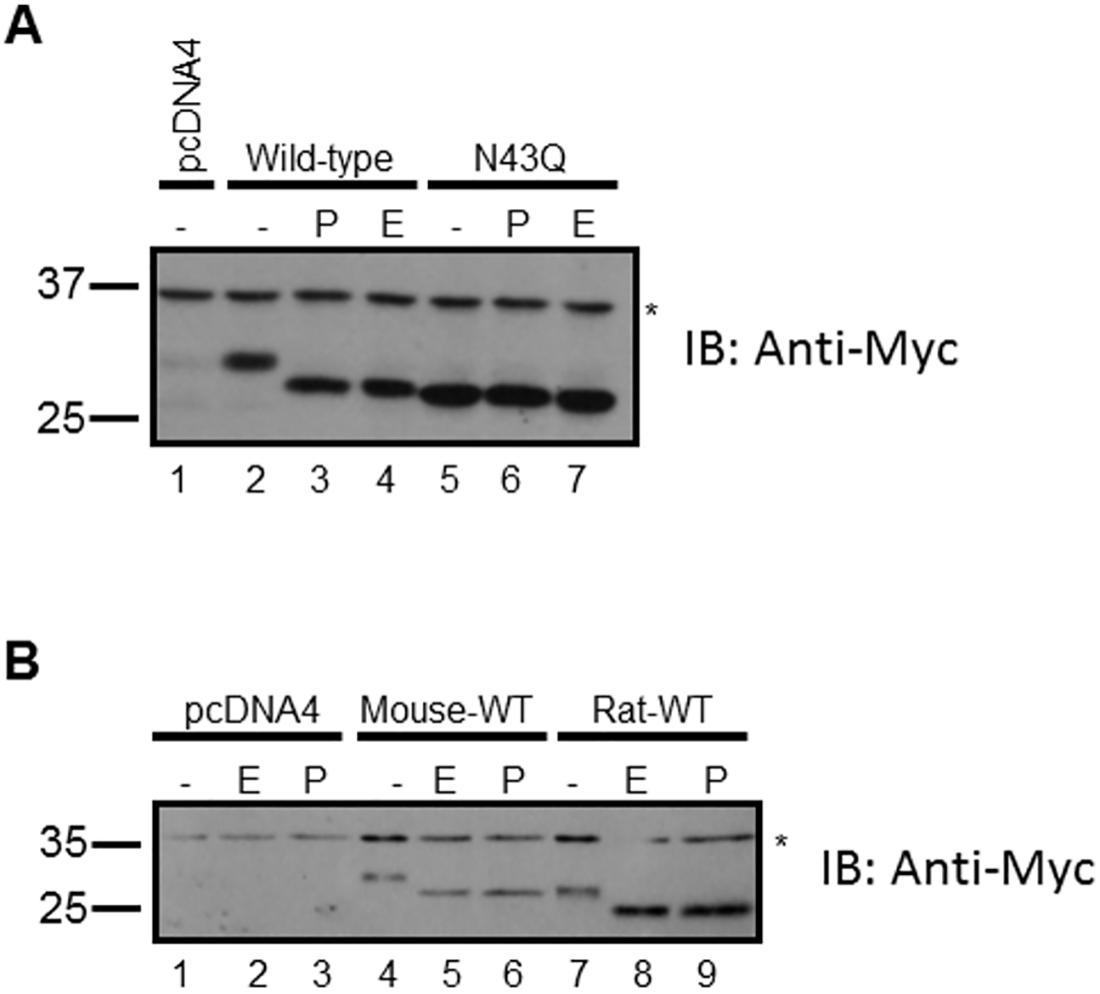
Murine and rat IGSF1-2 are glycoproteins. A) CHO cells were transfected with empty vector (pcDNA4), wild-type murine IGSF1-2-Myc/His, or murine IGSF1-2 (N43Q)-Myc/His expression vectors. Whole cell protein lysates were collected and treated with PNGaseF (P), EndoH (E), or buffer alone (-) before being subjected to SDS-PAGE and immunoblotting (IB) with a Myc antibody. Molecular weight markers (in kDa) are shown at the left. Lanes are numbered at the bottom. B) CHO cells were transfected and lysates analyzed as in panel A, with the following exception: wild-type rat IGSF1-2-Myc/His was used in place of the mutant murine expression vector. *, non-specific band.

To enable investigations of the IGSF1-2 protein isoform, we cloned the murine cDNA into an expression vector that added a Myc/His tag to the C-terminus. This was necessary as antibodies against the N-terminus of IGSF1 produced inconsistent results (data not shown). When expressed in CHO cells, murine IGSF1-2 migrated as a single protein species of ~28 kDa (Fig 1A, lane 2). The previously described IGSF1-NTD and—CTD are glycoproteins [11]. IGSF1-2 is similarly predicted to be glycosylated at an asparagine residue (Asn43) in the first of its 2 Ig loops. Consistent with this idea, removal of N-linked sugars with either PNGaseF or EndoH hastened migration of the protein on SDS-PAGE (Fig 1A; lanes 3 and 4). Moreover, mutation of Asn43 to Gln (N43Q) caused a similar increase in IGSF1-2’s mobility (lane 5), which was not further altered by PNGaseF or EndoH (lanes 6 and 7). Collectively, these data indicate that murine IGSF1-2 is a glycoprotein, which is glycosylated at Asn43. Moreover, the equivalent effects of EndoH and PNGaseF suggest that the protein only acquires immature sugars and may therefore not transit from the ER to the Golgi in the secretory pathway.

**Fig 2:**
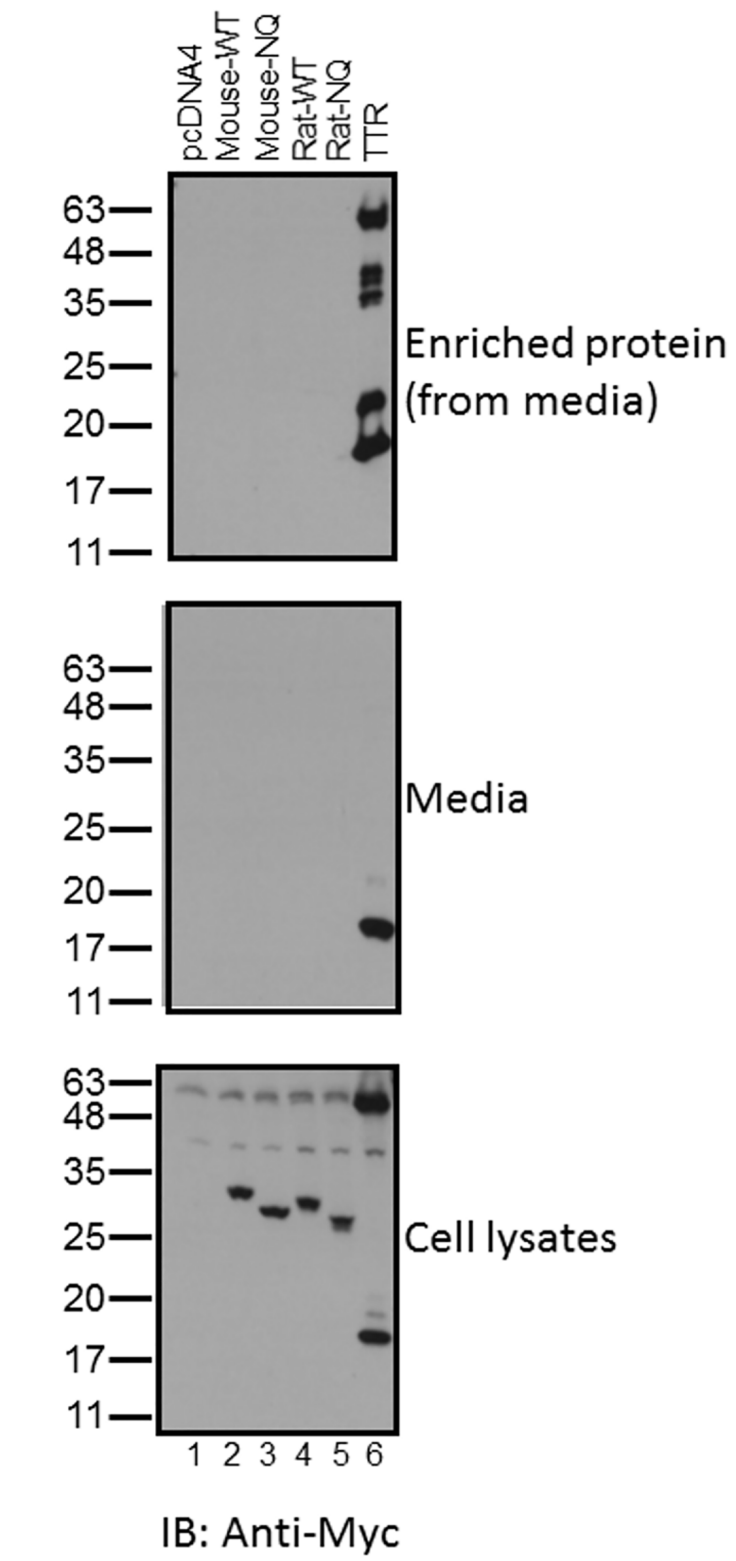
Murine and rat IGSF1-2 proteins are not secreted from transfected cells. HEK293 cells were transfected with expression plasmids for wild-type (WT) or glycosylation mutant (NQ) forms of murine or rat IGSF1-2, murine transthyretin (TTR), or empty vector (pcDNA4). Note, in all cases, proteins were expressed with Myc/His tags at their C-termini. Media (top two panels) and whole cell protein lysates (bottom panel) were collected and analyzed by SDS-PAGE and immunoblotting for Myc. In the middle panel, proteins in culture medium were analyzed directly. In the top panel, proteins in the media were analyzed following Ni-NTA purification and enrichment.

To assess the generality of these results, we repeated the analyses with rat IGSF1-2. Both murine and rat IGSF1-2 migrated as single protein species when expressed in CHO cells (Fig 1B, lanes 4 and 7). Again, treatment with EndoH or PNGaseF caused similar patterns of deglycosylation in both species (lanes 5, 6, 8, and 9). Comparable results were observed in a second cell line (HEK293; data not shown and Fig 2). Thus, IGSF1-2 is a glycoprotein containing only immature N-linked sugars in both mouse and rat. Interestingly, though the rat and murine IGSF1-2 proteins are of similar length (233 and 232 amino acids, respectively), the rat protein consistently migrated more rapidly on SDS-PAGE than the mouse (compare lanes 4 and 7 in Fig 1B). This likely resulted from differences in the cloning strategies for the expression constructs between the two species and differences in the lengths of their signal peptides (18 and 10 amino acids, respectively, according to SignalP-4.1).

Although IGSF1-2 was originally predicted to be secreted [10], the EndoH-sensitivity of the protein suggested that it might not exit the ER. We therefore asked whether IGSF1-2 could be detected in culture medium of transfected cells. HEK293 cells were transiently transfected with wild-type or glycosylation-deficient forms of murine (N43Q or NQ) or rat (N44Q or NQ) IGSF1-2 or the thyroid hormone binding protein transthyretin (TTR). The latter is a secreted protein [21] and was included as a positive control. As with IGSF1-2, the TTR expression vector included a C-terminal Myc/His tag. As demonstrated by immunoblotting of whole cell lysates, all proteins were expressed at roughly equivalent levels (Fig 2, lanes 2–6, bottom panel). In contrast, only TTR could be detected in culture medium, whether or not proteins were enriched via Ni-NTA chromatography (Fig 2, lane 6, middle and top panels). Equivalent results were observed in a second heterologous cell line (CHO; data not shown).

**Fig 3:**
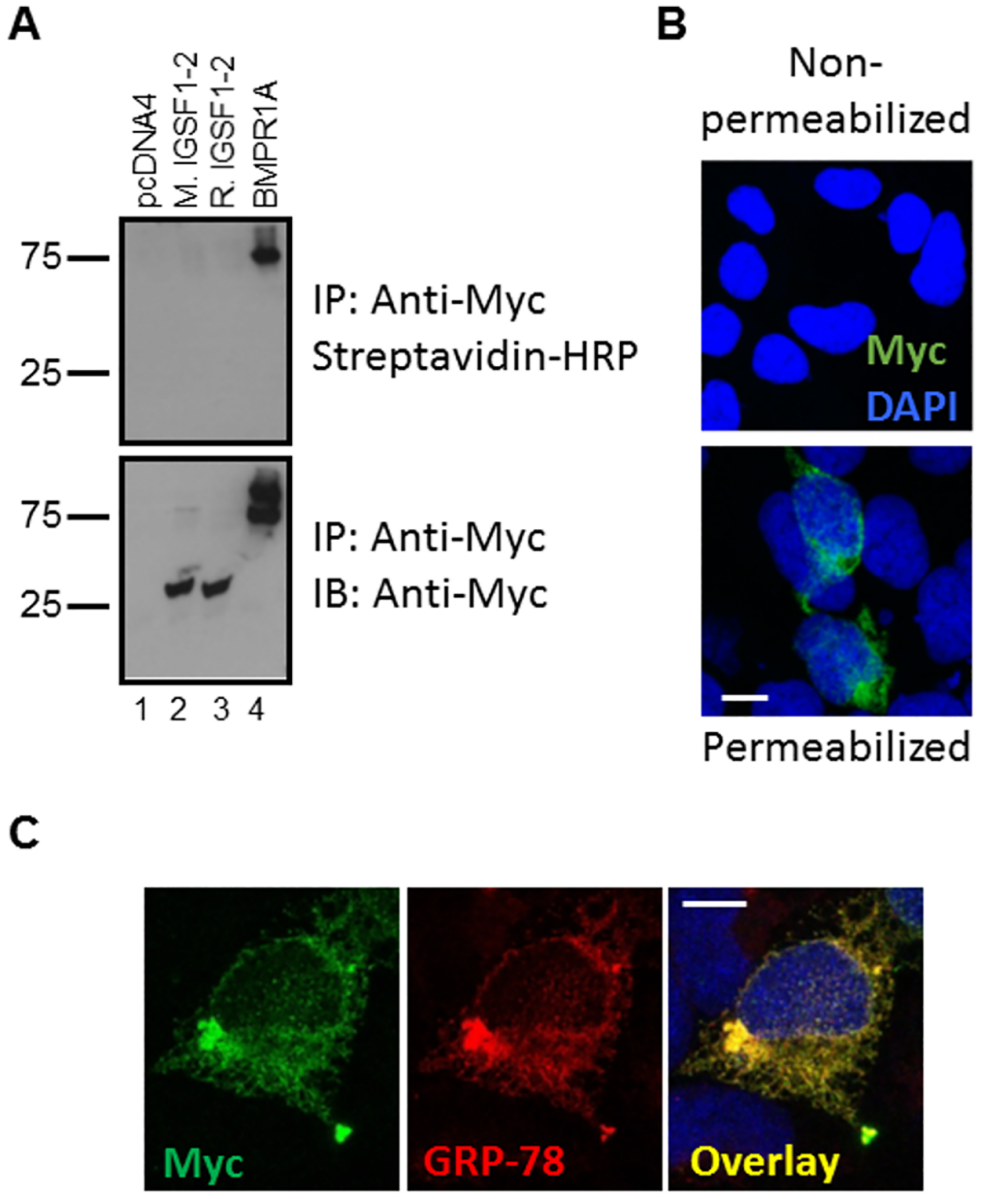
Murine and rat IGSF1-2 proteins do not traffic to the plasma membrane of transfected cells. A) HEK293 cells were transfected with expression plasmids for wild-type murine (M.) or rat (R.) IGSF1-2, murine BMP type IA receptor (BMPR1A), or empty vector (pcDNA4). Note, IGSF1-2 proteins were expressed with Myc/His tags at their C-termini, whereas BMPR1A had the Myc tag alone. Cell surface proteins were labeled with biotin prior to collection of whole cell lysates. Lysates were immunoprecipitated (IP) with Myc-beads and then subjected to SDS-PAGE and transferred to nitrocellulose membranes. Total proteins were immunoblotted with anti-Myc (bottom), whereas biotinylated proteins were identified with streptavidin-HRP (top). B) HEK293 cells were cultured on coverslips and transiently transfected with wild-type murine IGSF1-2-Myc/His. Cells were then fixed and subjected to immunofluorescence with a Myc antibody (green) under non-permeabilizing (top) and permeabilizing conditions (bottom). C) Cells were transfected as in panel B, premeabilized, and processed for double-label immunofluorescence with the Myc antibody (green) and an antibody against GRP-78/BiP (red). The overlay is shown in yellow. In B and C, nuclei were stained with DAPI. Images were captured by confocal microscopy. Scale bar, 10 μm.

Although these results suggested that IGSF1-2 was not secreted, they did not definitively show that the protein failed to transit out of the ER. For example, it is possible that the protein might navigate the secretory pathway, but remain associated with the plasma membrane. Though IGSF1-2 lacks a transmembrane domain or motif for GPI-linkage, this did not rule out association through protein-protein or other biophysical interactions. We therefore used cell-surface biotinylation to assess whether IGSF1-2 associates with the plasma membrane. HEK293 cells were transfected with C-terminally Myc-tagged forms of IGSF1-2 (mouse and rat) or the human BMP type IA receptor (BMPR1A). The latter is a transmembrane glycoprotein and was used as a positive control. Roughly equivalent amounts of the proteins were immunoprecipitated with a Myc antibody (Fig 3A, lanes 2–4, bottom panel). BMPR1A runs as a doublet, with the higher molecular weight band representing the mature, plasma membrane form of the protein. As shown in the cell-surface biotinylation analysis, only this form of the protein was detected at the plasma membrane (Fig 3A, lane 4, top panel). Neither murine nor rat IGSF1-2 was detected at the membrane (lanes 2 and 3, top panel). In complementary immunofluorescence analyses, IGSF1-2 protein could be detected in permeabilized, but not non-permeabilized cells (Fig 3B, compare top and bottom panels). Moreover, IGSF1-2 colocalized with GRP-78/BiP, a well-described luminal ER protein (Fig 3C). These results are consistent with the hypothesis that IGSF1-2 is not a secreted or plasma membrane-associated protein, but rather remains within the cell, most likely in the ER.

**Fig 4:**
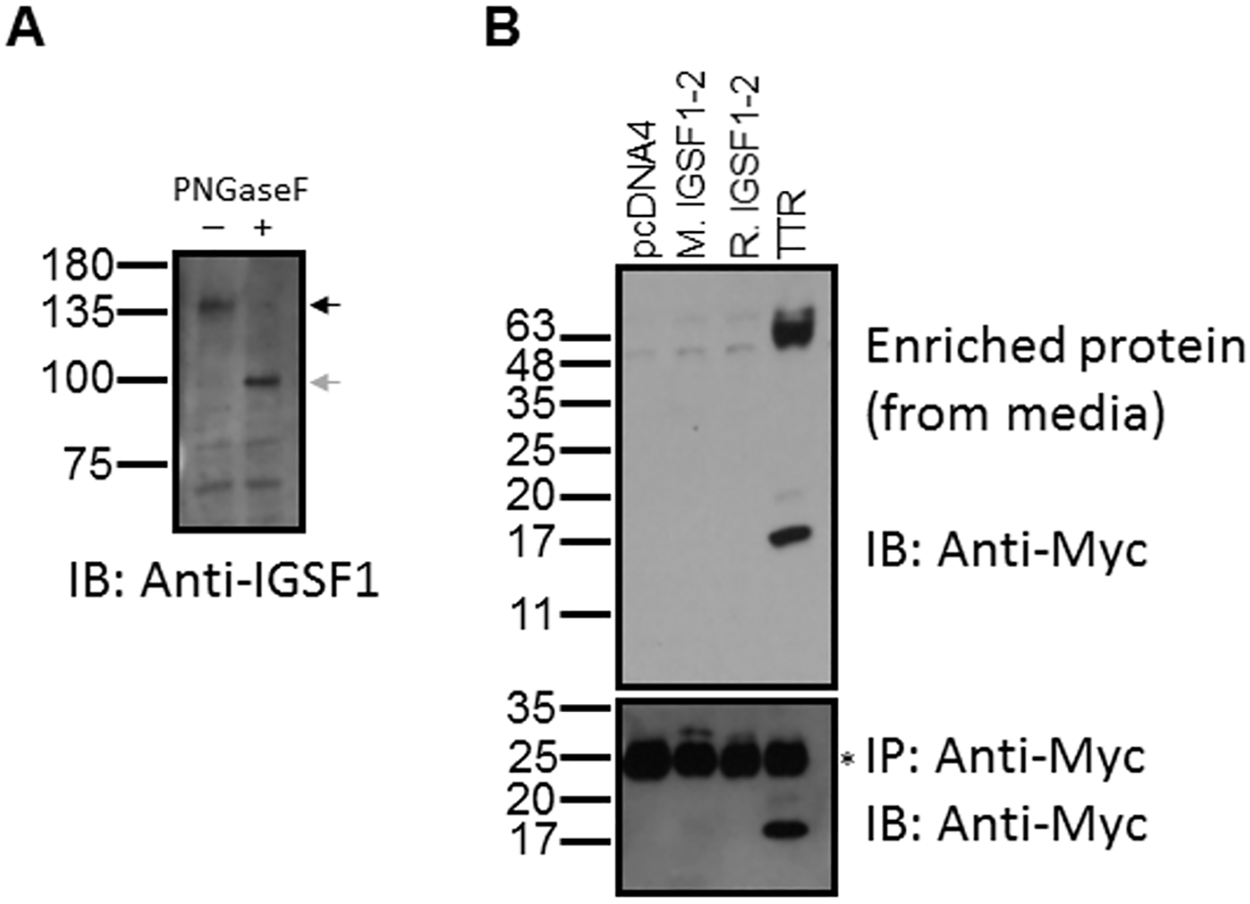
IGSF1-2 is not secreted from homologous HepG2 cells. A) Expression of endogenous IGSF1-CTD in HepG2 cells as determined by immunoblot using an antibody that recognizes the N-terminus of the CTD (black arrow). Protein lysates were treated with PNGaseF to confirm the specificity of the signal (gray arrow). B) HepG2 cells were transfected with expression plasmids for wild-type murine (M.) or rat (R.) IGSF1-2, murine transthyretin (TTR), or empty vector (pcDNA4). In all cases, proteins were expressed with Myc/His tags at their C-termini. Media was collected and His-tagged proteins purified by Ni-NTA chromatography before analysis by SDS-PAGE and immunoblotting for Myc (top). Cell protein lysates were collected and immunoprecipitated (IP) with Myc-beads before analysis by Myc immunoblot (bottom). Note that the non-specific band (*) partially obscures the rat IGSF1-2 in the bottom panel (third lane).

One caveat to the above results is that all the analyses were performed in heterologous cells (CHO or HEK293). It was therefore possible that mechanisms required for IGSF1-2 secretion were absent. Thus, we turned to a homologous cell system. IGSF1 is expressed in developing liver [5] and in hepatocellular carcinoma [22]. IGSF1-CTD is expressed in the liver cancer cell line HepG2 (Fig 4A). Unfortunately, we were unable to confirm expression of endogenous IGSF1-2 in HepG2 cells, as we lack antibodies against this isoform. Nonetheless, when we expressed murine or rat IGSF1-2 in HepG2 cells, we were again unable to detect the proteins in the culture medium, in contrast to TTR (Fig 4B, top panel). These data further support the interpretation that IGSF1-2 is not a secreted protein, at least not in the cell lines investigated here.

The IGSF1-2 protein is highly conserved in amino acid sequence across mammals. A BLASTP search of the murine IGSF1-2 sequence returned definitive alignments to IGSF1-2 orthologs (as opposed to full-length IGSF1) in 16 mammalian species. Sequence identity ranged from 77% (big brown bat) to 96% (rat), with 87% identity in human IGSF1-2. This level of conservation suggests a functional role for the protein. It is presently unclear, however, what this role might be in the pituitary or other tissues. It is possible that IGSF1-2 is secreted under conditions we were unable to recapitulate in cultured cells. As there is precedent for Ig superfamily members playing roles in the ER [23], we also cannot rule out intracellular functions. That said, we generated two *Igsf1*-deficient mouse models, which have similar phenotypes [3, 4, 7]. The first model [4] lacks multiple IGSF1 isoforms (including IGSF1-2), whereas IGSF1-2 is intact in the second model [7] (Brûlé and Turgeon, unpublished). This suggests that IGSF1-2 is not functional or that its function depends on the co-expression of other IGSF1 isoforms.

In summary, the 2 Ig loop isoform of IGSF1 (IGSF1-2) is highly expressed in the pituitary gland and is conserved across mammalian species. Despite earlier predictions [10], IGSF1-2 does not appear to be secreted. Therefore, any functions of the protein may be intracellular, perhaps in the ER. Most pathogenic mutations in *IGSF1* map to the portion of the gene encoding the CTD. As a result, IGSF1-2 should be intact in these individuals. In a few families, the entirety of the *IGSF1* gene is deleted; however, their phenotypes are similar to those of individuals harboring missense, nonsense, or frame-shift mutations in the CTD [3, 18]. Therefore, IGSF1-2 cannot compensate for the absence of the IGSF1-CTD. IGSF1-2’s function, like that of the CTD, remains to be determined.

## References

[1] Joustra SD, van Trotsenburg AS, Sun Y, Losekoot M, Bernard DJ, Biermasz NR, et al IGSF1 deficiency syndrome: A newly uncovered endocrinopathy. Rare Dis. 2013;1:e24883 doi: 10.4161/rdis.24883 ; 25002994 10.4161/rdis.24883 PMC3915563

[2] Joustra SD, Schoenmakers N, Persani L, Campi I, Bonomi M, Radetti G, et al The IGSF1 deficiency syndrome: characteristics of male and female patients. J Clin Endocrinol Metab. 2013;98(12):4942–52. doi: 10.1210/jc.2013-2743 . 24108313 10.1210/jc.2013-2743

[3] Sun Y, Bak B, Schoenmakers N, van Trotsenburg AS, Oostdijk W, Voshol P, et al Loss-of-function mutations in IGSF1 cause an X-linked syndrome of central hypothyroidism and testicular enlargement. Nat Genet. 2012;44(12):1375–81. doi: 10.1038/ng.2453 ; 23143598 10.1038/ng.2453 PMC3511587

[4] Bernard DJ, Burns KH, Haupt B, Matzuk MM, Woodruff TK. Normal reproductive function in InhBP/p120-deficient mice. Mol Cell Biol. 2003;23(14):4882–91. doi: 10.1128/MCB.23.14.4882-4891.2003 ; 12832474 10.1128/MCB.23.14.4882-4891.2003 PMC162213

[5] Joustra SD, Meijer OC, Heinen CA, Mol IM, Laghmani el H, Sengers RM, et al Spatial and temporal expression of immunoglobulin superfamily member 1 in the rat. J Endocrinol. 2015;226(3):181–91. doi: 10.1530/JOE-15-0204 . 26163525 10.1530/JOE-15-0204

[6] Joustra SD, Roelfsema F, Endert E, Ballieux BE, van Trotsenburg AS, Fliers E, et al Pituitary Hormone Secretion Profiles in IGSF1 Deficiency Syndrome. Neuroendocrinology. 2016;103(3–4):408–16. doi: 10.1159/000439433 . 26336917 10.1159/000439433

[7] Turgeon MO, Silander TL, Doycheva D, Liao XH, Rigden M, Ongaro L, et al TRH action is impaired in pituitaries of male IGSF1-deficient mice. Endocrinology. 2017;158(3). 10.1210/en.2016-1788 PMC5460797 28324000

[8] Frattini A, Faranda S, Redolfi E, Allavena P, Vezzoni P. Identification and genomic organization of a gene coding for a new member of the cell adhesion molecule family mapping to Xq25. Gene. 1998;214(1–2):1–6. . 9729118 10.1016/s0378-1119(98)00253-4

[9] Mazzarella R, Pengue G, Jones J, Jones C, Schlessinger D. Cloning and expression of an immunoglobulin superfamily gene (IGSF1) in Xq25. Genomics. 1998;48(2):157–62. doi: 10.1006/geno.1997.5156 . 9521868 10.1006/geno.1997.5156

[10] Bernard DJ, Woodruff TK. Inhibin binding protein in rats: alternative transcripts and regulation in the pituitary across the estrous cycle. Mol Endocrinol. 2001;15(4):654–67. doi: 10.1210/mend.15.4.0630 . 11266515 10.1210/mend.15.4.0630

[11] Robakis T, Bak B, Lin SH, Bernard DJ, Scheiffele P. An internal signal sequence directs intramembrane proteolysis of a cellular immunoglobulin domain protein. J Biol Chem. 2008;283(52):36369–76. doi: 10.1074/jbc.M807527200 ; 18981173 10.1074/jbc.M807527200 PMC2662301

[12] Nakamura A, Bak B, Silander TL, Lam J, Hotsubo T, Yorifuji T, et al Three novel IGSF1 mutations in four Japanese patients with X-linked congenital central hypothyroidism. J Clin Endocrinol Metab. 2013;98(10):E1682–91. doi: 10.1210/jc.2013-1224 . 23966245 10.1210/jc.2013-1224

[13] Van Hulle S, Craen M, Callewaert B, Joustra S, Oostdijk W, Losekoot M, et al Delayed Adrenarche may be an Additional Feature of Immunoglobulin Super Family Member 1 Deficiency Syndrome. J Clin Res Pediatr Endocrinol. 2016;8(1):86–91. doi: 10.4274/jcrpe.2512 ; 26757742 10.4274/jcrpe.2512 PMC4805054

[14] Joustra SD, Heinen CA, Schoenmakers N, Bonomi M, Ballieux BE, Turgeon MO, et al IGSF1 Deficiency: Lessons From an Extensive Case Series and Recommendations for Clinical Management. J Clin Endocrinol Metab. 2016;101(4):1627–36. doi: 10.1210/jc.2015-3880 ; 26840047 10.1210/jc.2015-3880 PMC4880178

[15] Joustra SD, Wehkalampi K, Oostdijk W, Biermasz NR, Howard S, Silander TL, et al IGSF1 variants in boys with familial delayed puberty. Eur J Pediatr. 2015;174(5):687–92. doi: 10.1007/s00431-014-2445-9 . 25354429 10.1007/s00431-014-2445-9

[16] Nishigaki S, Hamazaki T, Fujita K, Morikawa S, Tajima T, Shintaku H. A Japanese Family with Central Hypothyroidism Caused by a Novel IGSF1 Mutation. Thyroid. 2016;26(12):1701–5. doi: 10.1089/thy.2016.0005 . 27762734 10.1089/thy.2016.0005

[17] Tenenbaum-Rakover Y, Turgeon MO, London S, Hermanns P, Pohlenz J, Bernard DJ, et al Familial Central Hypothyroidism Caused by a Novel IGSF1 Gene Mutation. Thyroid. 2016 doi: 10.1089/thy.2015.0672 . 27310681 10.1089/thy.2015.0672

[18] Hughes JN, Aubert M, Heatlie J, Gardner A, Gecz J, Morgan T, et al Identification of an IGSF1-specific deletion in a five-generation pedigree with X-linked Central Hypothyroidism without macroorchidism. Clin Endocrinol (Oxf). 2016;85(4):609–15. doi: 10.1111/cen.13094 . 27146357 10.1111/cen.13094

[19] Tanaka S, Tatsumi K, Okubo K, Itoh K, Kawamoto S, Matsubara K, et al Expression profile of active genes in the human pituitary gland. J Mol Endocrinol. 2002;28(1):33–44. . 11854097 10.1677/jme.0.0280033

[20] Ho CC, Bernard DJ. Bone morphogenetic protein 2 signals via BMPR1A to regulate murine follicle-stimulating hormone beta subunit transcription. Biol Reprod. 2009;81(1):133–41. doi: 10.1095/biolreprod.108.074211 ; 19211807 10.1095/biolreprod.108.074211 PMC3093989

[21] Alshehri B, D'Souza DG, Lee JY, Petratos S, Richardson SJ. The diversity of mechanisms influenced by transthyretin in neurobiology: development, disease and endocrine disruption. J Neuroendocrinol. 2015;27(5):303–23. doi: 10.1111/jne.12271 . 25737004 10.1111/jne.12271

[22] Patil MA, Chua MS, Pan KH, Lin R, Lih CJ, Cheung ST, et al An integrated data analysis approach to characterize genes highly expressed in hepatocellular carcinoma. Oncogene. 2005;24(23):3737–47. doi: 10.1038/sj.onc.1208479 . 15735714 10.1038/sj.onc.1208479

[23] Cabrera CM. The double role of the endoplasmic reticulum chaperone tapasin in peptide optimization of HLA class I molecules. Scand J Immunol. 2007;65(6):487–93. doi: 10.1111/j.1365-3083.2007.01934.x . 17523940 10.1111/j.1365-3083.2007.01934.x

